# Health-Related Quality of Life Outcomes from Botulinum Toxin Treatment in Spasticity

**DOI:** 10.3390/toxins12050292

**Published:** 2020-05-04

**Authors:** Lorenzo Pietro Roncoroni, Daniel Weiss, Leonhard Hieber, Justine Sturm, Axel Börtlein, Ingo Mayr, Matthias Appy, Benedicta Kühnler, Joachim Buchthal, Christian Dippon, Guy Arnold, Tobias Wächter

**Affiliations:** 1 Hertie-Institute for Clinical Brain Research, Department of Neurodegenerative Diseases, 72076 Tübingen, Germany; lorenzo.roncoroni@arcor.de (L.P.R.); leonhard.hieber@gmx.de (L.H.); justine.sturm@googlemail.com (J.S.); 2 Department of Neurodegenerative Diseases, Centre of Neurology, University of Tübingen, 72076 Tübingen, Germany; 3 Neurologische Klinik, Klinikum Stuttgart, 70174 Stuttgart, Germany; ABoertlein@klinikum-stuttgart.de; 4 Klinik für Neurologie Sindelfingen, Krankenhaus Sindelfingen-Böblingen, 71065 Sindelfingen, Germany; I.Mayr@klinikverbund-suedwest.de (I.M.); G.Arnold@klinikverbund-suedwest.de (G.A.); 5 Berufsausübungsgemeinschaft Dres, Matthias Appy, Wolfgang Molt, Prof. Arthur Melms und Kollegen, 70176 Stuttgart, Germany; appy@neurologen-stuttgart.de (M.A.); kuehnler@neurologen-stuttgart.de (B.K.); 6 Neurologische Gemeinschaftspraxis am Seelberg, 70372 Stuttgart, Germany; joachim.buchthal@gmx.de (J.B.); drcdippon@gmx.de (C.D.); 7 Centre of Movement Disorders and Centre of Rehabilitation PASSAUER WOLF, 93333 Bad Gögging, Germany

**Keywords:** spasticity, paresis, dystonia, botulinum toxin, quality of life

## Abstract

Objective: The effects of botulinum toxin injections (BoNT) on health-related quality of life along the complex spectrum of spasticity needs further characterization to guide practitioners in a real-life therapeutic environment. Methods: In this study, we analyzed 50 consecutive and unselected patients with spasticity before and four weeks after re-injection of botulinum toxin. Health-related quality of life in terms of the EuroQol (EQ) as well as further motor and non-motor characteristics were assessed. Results: BoNT improved the EQ visual analog scale (EQ VAS). In addition, state of health and pain maxima improved. The EQ VAS improvement correlated with pre-injection characteristics of the EQ VAS and life satisfaction in the “movement disorders” domain. Conclusion: EQ VAS is sensitive for monitoring HR-QoL outcomes in an unselected real life observational cohort. This study may inform future studies intended to validate prediction variables that could inform on HR-QoL effects of BoNT treatment in spasticity.

## 1. Introduction

Spasticity occurs in a diverse set of etiologies including ischemic stroke, traumatic brain injury, autoimmune inflammatory disease, hypoxia, and others. Spasticity may deteriorate health-related quality of life (HR-Qol) through different mechanisms. Impairments in mobility, loss of self-dependence and need of nursing support, anxiety, and depression may reduce the self-perceived state of health and satisfaction in life [1,2]. In addition, recent studies identified additional modifiers of self-perceived HR-Qol in spasticity; one study in 62 children with cerebral palsy reported that spasticity affected gross motor functions [3], and that impaired motor function significantly deteriorated HR-Qol [3]. Another study identified that pain associated with spasticity modified HR-Qol [4]. It should be emphasized that pain related to spasticity is reported with a high prevalence in these cohorts, e.g., 65% in a recent study [1]. Another more systematic review on post-stroke leg spasticity concluded that gait performance improved with BoNT treatment. However, this did not result in improved HR-Qol [5]. A larger meta-analysis on HR-Qol was not possible owing to the small number of well-controlled trials [6]. Given that the etiological spectrum of spasticity and its distribution is highly variable across subjects and cohorts, an analysis embracing several etiologies and phenotypes would essentially benefit practitioners in real-world therapeutic settings in order to make decisions about BoNT therapy for spasticity. In addition, it may be challenging to evaluate whether patients with ongoing BoNT therapy benefit from the injections with respect to HR-QoL.

In this study, we therefore aimed to characterize the therapeutic outcomes of patients treated with ongoing BoNT therapy for spasticity in a real-world setting. In particular, we were interested (i) to learn whether BoNT treatment is effective on HR-Qol in an unselected cohort of patients with spasticity; (ii) which motor and non-motor domains improve with BoNT; and (iii) which “pre-injection” motor and non-motor characteristics associate with HR-QoL outcomes.

## 2. Results

Patients reported improvement in HR-QoL according to the EQ VAS (detailed statistical descriptions are given in Table 1). EQ-5D-5L showed a slight mean improvement, although this did not reach significance. State of health improved after BoNT injection. Pain maxima were reduced. Average pain was slightly reduced, although this did not reach significance. Overall sleep quality in terms of overall PSQI changed slightly. Life satisfaction domains (FLZ-A, FLZ-G, FLZ-BS), BDI, SIS 16 and the PSQI single domain components 1 to 7 showed no significant changes between the two time-points (Table 1).

In order to better understand the mechanisms of EQ VAS improvement from BoNT, we correlated the change of EQ VAS between Time-point 1 and Time-point 2 with the changes in other variables that showed significant difference between Time-point 1 and Time-point 2. There was no significant correlation between the difference of EQ VAS and the differences in either state of health, maximum pain, or overall PSQI (Table 2).

Next, we wished to gain insight as to whether the patient characteristics at Time-point 1 associated with EQ VAS improvement. To this end, we correlated the change in EQ VAS as difference between Time-point 1 and Time-point 2 with the variables at Time-point 1. We found significant correlations of EQ VAS change with “EQ VAS” and FLZ-BS (life satisfaction—movement disorders domain) at Time-point 1 (Table 3), indicating that higher impairment in EQ VAS at Time-point 1 correlated with better improvement from BoNT injection.

## 3. Discussion

This study prospectively evaluated the effect of BoNT therapy on HR-QoL in an unselected cohort of patients with spasticity in a real-world therapeutic environment. BoNT therapy improved the EQ VAS, but also the state of health and pain maxima. These changes were statistically significant, although the differences were within the range of the results were small. 

Quality of life improved on the non-specific and more fine-grained EQ VAS scale, whereas the EQ-5D-5L, which refers more concretely to direct assessments of self-estimated mobility, self-care, usual activities, pain/discomfort, and anxiety/depression compared to the EQ VAS, did not show a significant improvement. One may argue that the impairments in these domains might indicate some heterogeneity across spasticity subtypes and therefore explain the lack of a group-level effect in this unselected cohort. Instead, subpopulations may more directly attribute their individual symptoms to a visual analog scale. It must also be noted that the effect of the improvement in the EQ VAS and in the pain maxima was relatively small. EQ VAS therefore seems be more sensitive to detect the HR-QoL changes in BoNT therapy, and it could be subject to future studies to evaluate whether this could be complemented by more disease- and symptom-specific scales.

Recent studies on BoNT therapy [6,7] focused on specific functional improvement but seldom on its implication for HR-QoL. Two such studies have demonstrated a beneficial effect of BoNT treatment on upper limb spasticity, but this did not transfer into HR-QoL improvement [8,9]. In addition, two different studies showed a functional benefit of BoNT injection on lower limb function, again missing a transfer to HR-QoL improvement [5,10]. On the one hand, studies examining the effect of BoNT treatment on spasticity of only one location and one etiology would allow more precise attributions of the clinical effect of BoNT therapy. However, the quality of life effects may be less specific and attributable and more complex in nature, as suggested by our results. So far, only one study [11] has examined the effect of BoNT treatment on spasticity independent from etiology and distribution in a design similar to our study. Interestingly, this study also found an unspecific, but significant effect on life quality in the areas of social and physical function [11].

It was noteworthy that there was also an improvement in pain maxima and state of health. Based on previous findings, pain maxima in particular could be expected to be the main determinant of HR-QoL impairments [4]. Interestingly however, EQ VAS improvement and pain maxima improvement were uncorrelated. This is not to conclude that pain maxima are irrelevant to EQ VAS. However, it is probable, that the contributions to EQ VAS are diverse and therefore would not correlate as a single variable. The clinical heterogeneity of spasticity distribution and the variable influence of pain among these subtypes would be an alternative interpretation.

An interesting finding of this study is that a few variables at Time-point 1 correlated to HR-QoL improvement. This mainly included EQ VAS at Time-point 1 and also satisfaction in the life “movement disorders” domain, which quantifies the relevance of movement disorder symptoms for daily life satisfaction and functionality. It seems an important note that the self-perceived impairment and loss of quality of life and life satisfaction are critical to the experience of a self-perceived benefit from the therapy. In other words, patients with more severe deteriorations may have more to gain. This finding is well in line with recent findings on HR-QoL prediction in movement disorder disciplines, for example in deep-brain stimulation therapy for Parkinson’s disease [12]. To this end, it seems most critical for practitioners to evaluate carefully whether and how spasticity affects the HR-QoL of an individual patient when making decisions about BoNT (especially its continuation). The pre-injection characteristics of patients under therapy may guide the stratification of patients and improve the chance for treatment success, particularly in those patients who experience a relevant deterioration of EQ VAS and life satisfaction at the end of a 3 month re-injection interval.

## 4. Limitations

This study was performed as prospective study. BoNT treatment was delivered by several well-experienced neurologists, but it was not placebo-controlled or blinded. Therefore, all subjects knew that the treatment was deemed effective for their functional deficit. Furthermore, the ratings were all self-evaluations done by the patient at the day of injection and four weeks after the treatment. The ratings therefore might be influenced by the expectations of the patients. Benefits on quality of life in patients with very high expectations might have been understated in their ratings. It must also be stated that subjects received BoNT treatment for a longer period in time prior to the study. The treatment period varied between subjects. This might lead to different effects, which were neither controlled nor analyzed in this study. Furthermore, for clinical and ethical reasons, the washout period of BoNT therapy lasted only for three months in this study. Longer-lasting therapeutic effects of BoNT cannot be excluded and, therefore, the ratings at Time-point 1 may have underestimated the true quality of life impairments if therapy were to be discontinued for longer. The design of the study unfortunately did not allow the effect of the role of the injection regime on the outcome to be verified, which is a further limitation of the study. Our intention was to study a diverse cohort of patients treated in clinical settings at different institutions, with different etiologies and a wide range of ages. Therefore, this cohort was randomly selected and represented a variety of clinical representations of spasticity. Because of the heterogeneity of the patients, the results must be interpreted with caution. It must also be noted that because of the data acquisition during clinical routines, the information concerning the clinical characteristics was incomplete in 18 patients. This is important to take into account, especially since the observed improvement in the EQ-VAS observed was relatively small. 

## 5. Conclusions

In a population of patients with spasticity of different etiology and distribution, treatment with BoNT led to a significant benefit on quality of life on the EQ VAS. Furthermore, the gain in EQ VAS was paralleled by suppression of pain maxima, although not correlated. Moreover, severe impairments on HR-QoL prior to the pre-injection point led to more favorable treatment successes. Further prospective studies in larger populations are needed to further evaluate the predictive value of these variables on treatment success of BoNT for spasticity. As this study demonstrated an improvement of quality of life by the treatment with BoNT in patients with spasticity that did not correlate with other clinical parameters, it seems important to include quality of life scales as outcome parameters in future studies of BoNT treatment in patients with spasticity.

## 6. Materials and Methods

This report on HR-Qol outcomes in patients with spasticity was part of a larger observational study that reported outcomes before and after re-injections of BoNT treatment in different established and approved indications of BoNT treatment. Findings in cervical dystonia [13], blepharospasm [14], and hemifacial spasm [15] are reported elsewhere.

### 6.1. Patients

A total of 63 patients with spasticity provided written informed consent to participate in this observational study. Of these, 13 patients did not participate in any assessments, and were therefore excluded from further analyses. The remaining 50 patients (patient characteristics in Table 4) with spasticity of different etiology and distribution (clinical characteristics in Table 5) and ongoing BoNT therapy (47 re-injections, three patients with first-time injection) were used for further analyses. The following etiologies of spasticity were represented in this study: ischemic stroke, intracranial bleeding, early childhood brain damage, multiple sclerosis, spinal pathology, hypoxic brain damage, and traumatic brain lesions. Mean patient age at the time-point of giving informed consent was 54.8 ± 15.2 years, with a mean disease duration of 12.3 ± 14.2 years.

### 6.2. Study Design

The study was performed at the end of a regular 3 month washout period from last injection prior to re-injection (Time-point 1) and four weeks after re-injection of BoNT (Time-point 2). Botulinum preparations included AbobotulinumtoxinA (n = 14), IncobotulinumtoxinA (n = 14), OnabotulinumtoxinA (n = 7). One patient received both IncobotulinumtoxinA and BotulinumtoxinB (n = 1). The preparations were not reported in 14 patients. Time-point 1 was scheduled twelve weeks from last injection. Optimal BoNT efficacy was expected four weeks after injection at Time-point 2. Inclusion criteria were: clinically diagnosed spasticity and age over 18 years. Patients on anticoagulants were excluded. 

The study was approved by the local ethics committee of Tuebingen University (516/2011BO2) and Landesärztekammer Baden-Württemberg. All patients provided written informed consent. Inclusion and treatment of patients was performed at the Centre for Neurology, Department for Neurodegenerative Diseases, Tübingen University, the Centre for Neurology at the Bürgerhospital Stuttgart, the neurology practices “Dr. Appy & Molt” and “am Seelberg” in Stuttgart, and the Centre of Neurology in Sindelfingen.

### 6.3. Assessments

In this study, we were interested in the effect of BoNT therapy on the quality of life in patients with spasticity. We therefore assessed as our primary outcome general quality of life (EQ-5D-5L including the EQ-VAS). As a secondary outcome, we assessed factors that could influence life quality, in particular global state of health (short Form Health Survey) life satisfaction (FLZ), depression (Beck Depression Scala), affected motor function (stroke impact scale), pain (visual analog scales), and sleep (Pittsburgh sleep quality index), as described in detail below. 

The scores were obtained in the German language. We evaluated the outcome of the EuroQol as main interest in this study. The EuroQol group developed a standardized non-disease-specific instrument called EQ-5D-5L [16] (EuroQol in five dimensions scaled in five levels of severity) to describe and quantify health-related quality of life; in the first section, EQ-5D1 inquires subjectively perceived mobility, self-care, usual activities, pain/discomfort, and anxiety/depression on a five point scale. Additionally, EQ VAS consists of a vertical visual analog scale from 0 (worst) to 100 (best) in order to assess self-rated momentary health state.

In addition, further scores were obtained as secondary interests. The patients’ global subjective impressions (“state of health”) were assessed in seven subdivisions, asking about improvement or worsening compared to four weeks before. The questionnaire on life satisfaction FLZ [17] (“Fragen zur Lebenszufriedenheit”), validated in the German language, included aspects of general life satisfaction (FLZ-A), satisfaction with health (FLZ-G), and movement disorders (FLZ-BS) (acronyms inspired by German language). Using the Beck’s Depression Inventory [18] (BDI) patients rated their overall impression of the last two weeks. The stroke impact scale [19] (SIS 16) including 16 items was used for assessment of impairment severity in the last two weeks. Pain assessment was performed using two horizontal visual analog scales reaching from the left corner (corresponding to 0 = no pain) to the right corner (corresponding to 10 = maximum pain). The first scale represented the worst pain perceived over the last week (maximum pain). The second scale represented the average pain level over the last week (average pain). Pittsburgh sleep quality index [20] (PSQI) was composed of ten questions, which were combined to seven “components” C1–7. Addition of the scores of C1–7 resulted in the overall PSQI score.

### 6.4. Statistical Analyses

We analyzed EuroQol as primary outcome measure. Distribution of the data was evaluated applying Shapiro–Wilk test. Based on the distribution, we used parametric t-test in case of normal distribution or non-parametric Wilcoxon tests to compare between Time-point 1 and Time-point 2. Pairwise comparisons were decided on a two-tailed significance level of *p* < 0.05. Significant changes in HR-Qol were considered for Spearman’s correlation analysis with other motor or non-motor variables. First, we correlated the change in EQ VAS between Time-point 1 and Time-point 2 with the change in other variables between Time-point 1 and Time-point 2 that showed significant difference in the pairwise comparison. Second, we correlated the change in EQ VAS with the Time-point 1 characteristics. All statistical analyses including the secondary variables were performed using SPSS version 25 (IBM), and with exploratory intent. This means that we did not adjust the *p*-values for multiple comparison, but intended that these findings would rather generate hypotheses for future studies, that could be verified in independent studies [21].

### 6.5. Ethics Statement

The study was approved by the Ethics Committee of Tübingen University (516/2011BO2) on the 8 December 2011 and Landesärztekammer Baden-Württemberg on the 10 January. 2012. All patients participated after providing written informed consent.

## Figures and Tables

**Table 1:** Pairwise comparisons between Time-point 1 and Time-point 2.

Assessment	Time-Point 1 (Mean ± SD/ Median (IQR))	Time-Point 2 (Mean ± SD/ Median (IQR))	Significance (*t*-Test)	T-Value	Significance (Wilcoxon Test)	Z-Value
State of health *	4 (1)	5 (2)			<0.001 *	−3.608
EQ-5D-5L	9.39 ± 5.14	8.96 ± 4.917	0.248	1.171		
EQ VAS *	46.09 ± 22.80	52.85 ± 23.90	0.025 *	−2.316		
FLZ A	29.35 ± 35.15	35.84 ± 30.81	0.144	−1.490		
FLZ G	15.00 ± 47.64	19.07 ± 46.89	0.413	−0.827		
FLZ BS	6 (92)	19 (84)			0.990	−0.13
BDI	12.84 ± 8.57	12.36 ± 8.29	0.573	0.568		
SIS 16	48 (26)	44 (32)			0.844	−0.197
Maximum pain *	2 (7)	2 (5)			0.037 *	−2.089
Average pain	2 (6)	2 (4)			0.351	−0.933
Overall PSQI *	6.35 ± 4.02	7.39 ± 4.44	0.037 *	−2.145		

EQ-5D-5L = subjectively perceived mobility, self-care, usual activities, pain/discomfort, and anxiety/depression on a five point scale; EQ VAS = vertical visual analog scale from 0 (worst) to 100 (best) to assess self-rated momentary HR-QoL; maximum pain = worst pain perceived over the last week on an horizontal visual analog scale reaching from the left corner (0 = no pain) to the right corner (10 = maximum pain); FLZ = Fragen zur Lebenszufriedenheit (questions about life satisfaction); A = general domain; G = health domain; BS = movement disorders domain; BDI = Beck’s Depression Inventory; SIS = stroke impairment scale; PSQI = Pittsburgh sleep quality index; SD = standard deviation; * pairwise comparisons significant at two-sided significance level of *p* < 0.05. Descriptive characteristics are reported as mean ± standard deviation in case of normal distribution, or as median (interquartile range) in case of non-normal distribution.

**Table 2:** Correlation between change in EQ VAS and change in other variables.

Correlations	Spearman’s Correlation
∆ EQ VAS–∆ maximum pain	r = −0.033; *p* = 0.831
∆ EQ VAS–∆ state of health	r = 0.194; *p* = 0.213
∆ EQ VAS–∆ overall PSQI	r = 0.089; *p* = 0.557

Here, we correlated the change in EQ VAS between Time-point 2 and Time-point 1 with the change in other variables between Time-point 2 and Time-point 1 that showed significant difference in the pairwise comparisons. There were no significant correlations between EQ VAS improvement and improvements of other variables. r = correlation coefficient; *p*: two-tailed significance level set to *p* < 0.05). PSQI = Pittsburgh sleep quality index.

**Table 3:** Correlations between EQ VAS change and other variables at Time-point 1.

T1 Variable in Correlation to EQ VAS Change	Correlation Coefficient	Significance Probability
State of health	r = −0.99	*p* = 0.523
EQ-5D-5L	r = −0.036	*p* = 0.813
EQ VAS	r = −0.341	*p* = 0.020
FLZ A	r = 0.297	*p* = 0.050
FLZ G	r = 0.148	*p* = 0.326
FLZ BS	r = 0.324	*p* = 0.030
BDI	r = −0.023	*p* = 0.881
SIS 16	r = 0.049	*p* = 0.750
Maximum Pain	r = −0.046	*p* = 0.762
Average Pain	r = −0.88	*p* = 0.563
Overall PSQI	r = −0.146	*p* = 0.333

Abbreviations: r = Spearman’s correlation coefficient; *p* = two-sided significance level at *p* < 0.05. EQ-5D-5L = subjectively perceived mobility, self-care, usual activities, pain/discomfort, and anxiety/depression on a five point scale; EQ VAS = vertical visual analog scale from 0 (worst) to 100 (best) to assess self-rated momentary health state; FLZ = life satisfaction, A = general domain, G = health domain, BS = movement disorders domain; BDI = Beck’s Depression Inventory; SIS = stroke impact scale; PSQI = Pittsburgh sleep quality index.

**Table 4:** Patient characteristics.

Item	Details	Details (Botulinum Preparations)	
Female:male	22:28		
Age at Time-point 1	54.8 ± 15.2 years		
Age at onset	46.1 ± 24.2 years		
Disease duration	12.3 ± 14.2 years		
First-time injection	N = 3		
Repeated injections	N = 47		
	Preparation	Number of patients	Dosage
Botulinum preparations (patients) (units ± SD)	AbobotulinumtoxinA	14	926 ± 419
	IncobotulinumtoxinA	14	203 ± 167
	OnabotulinumtoxinA	7	223 ± 87
	IncobotulinumtoxA + BotulinumtoxinB	1	275
	Missing information	14	n.a.

Abbreviations: SD = standard deviation.

**Table 5:** Etiology and distribution of spasticity.

Item	Specification	Number of Patients
**Etiology**	Spasticity, unspecified	18
	Ischemic stroke	9
	Intracranial bleeding	6
	Early childhood brain damage	5
	Multiple sclerosis	4
	Spinal pathology, unspecified	3
	Hypoxic brain damage	3
	Traumatic brain lesion	2
**Distribution of spasticity**	Focal spasticity arm unilateral leg	8 3 5
	Hemispasticity left right laterality unspecified	21 10 9 2
	Bilateral leg spasticity	8
	Tetraspasticity	9
	not specified	4
